# Therapeutic cancer vaccine: phase I clinical tolerance study of Hu-rhEGF-rP64k/Mont in patients with newly diagnosed advanced non-small cell lung cancer

**DOI:** 10.1186/s12865-018-0249-9

**Published:** 2018-04-16

**Authors:** Puyuan Xing, Hongyu Wang, Sheng Yang, Xiaohong Han, Yan Sun, Yuankai Shi

**Affiliations:** 1 Department of Medical Oncology, Beijing Key Laboratory of Clinical Study on Anticancer Molecular Targeted Drugs, National Cancer Center/Cancer Hospital, Chinese Academy of Medical Sciences & Peking Union Medical College, Beijing, 100021 China; 2 Department of Clinical Laboratory, National Cancer Center/Cancer Hospital, Chinese Academy of Medical Sciences & Peking Union Medical College, Beijing, 100021 China

**Keywords:** Cancer vaccine, Lung cancer, Clinical study

## Abstract

**Background:**

Hu-rhEGF-rP64k/Mont is a biotechnology product for the treatment of advanced non-small cell lung cancer (NSCLC). The vaccine induces a neutralizing antibody-mediated immune response, against the normal circulating self-protein antigen epidermal growth factor (EGF), which prevents its binding to and activation of the EGF receptor, inhibiting the transduction of the signals that drive cancer cell proliferation, survival and spread. This phase I study aimed to evaluate the safety and the immunological response of Hu-rhEGF-rP64k vaccine in NSCLC patients.

**Results:**

The Hu-rhEGF-rP64k/Mont vaccine showed to be safe and well tolerated, with dizziness, injection-site reactions and tremors being the most commonly reported adverse event. No severe adverse events or death were related to the vaccination. Immune monitoring demonstrated the generation of anti-EGF antibody titers and as a consequence the patients exhibited a decrease in the EGF concentration. In 80% of the vaccinated patients stable disease was achieved.

**Conclusion:**

Hu-rhEGF-rP64k/Mont elicited a valuable immune response, with good safety profile assuring further clinical development of the vaccine in this population to further confirm the potential benefits on survival.

**Trial registration:**

Chinese Clinical Trial Registry, ChiCTR-OID-17014048, date 2017/12/20 (retrospectively registered); Chinese Food and Drug Administration, CFDA 2009 L02105, date 2009/04/03; China Drug Trial, CTR20131039.

## Background

Lung cancer is the most common worldwide cancer, with 1.8 million new cases in 2012 (12.9% of the total). It is the most common cancer in men worldwide (1.2 million, 16.7% of the total), reaching rates of 50.4 per 100,000 in Eastern Asia and relatively high rates in women in this geographic region although the tobacco is generally rare in these population. The disease is the leading causes of cancer-related death around the world, responsible for the deaths by cancer in one every five cases of cancer (1.59 million deaths, 19.4% of the total) [1]. Non-small cell histology represents between 75% and 80% of lung tumors. More than 50% of cases are diagnosed in patients with local regional advanced or metastatic disease [2, 3].

The platinum-based doublet chemotherapy has been the standard first-line treatment for non-selected patients with advanced NSCLC [4] and only produce modest survival improve compared with best supportive care. The 1-year survival rate is approximately 35% for patients with wild type tumors whereas 5-year survival rate is still around 5% [5]. There is a great need for novel and more effective treatments for advanced NSCLC.

Hu-rhEGF-rP64k/Mont is a biotechnology product for the treatment of advanced NSCLC. It is a therapeutic vaccine composed by an antigen (recombinant human EGF (rhEGF) chemically conjugated to recombinant P64K (rP64K)) and an adjuvant (Montanide ISA51VG), developed by BIOTECH PHARMA Co., Ltd. and the Center of Molecular Immunology (Havana, Cuba). The antigen is obtained from the chemical conjugation using glutaraldehyde as a linker reagent of drug substance intermediates, the recombinant protein rhEGF, and the recombinant protein rP64K. Hu-rhEGF-rP64k/Mont cancer vaccine can induces a neutralizing antibody-mediated immune response, against the normal circulating self-protein antigen Epidermal Growth Factor (EGF), which is the main ligand of the Epidermal Growth Factor Receptor (EGF-R). This will inhibit the growth of tumor cells and vessels nourishing and lead to the apoptosis of tumor cells. Pre-clinical research results indicated this vaccine is immunogenic, well tolerated and has anti-tumor activity, especially for the NSCLC [6].

The clinical experience in advanced stage (IIIB/IV) NSCLC had demonstrated that the vaccine is very immunogenic, reducing the EGF concentration and increasing the anti-EGF antibody titers and it has been very well tolerated [7–11]. With the purpose of confirming the findings of previous studies, which have been conducted in Western population and considering that the management of NSCLC in China has differences in medical care, drug approval requirements, ethnic variation and clinical behavior we designed this early stage clinical trial in Chinese NSLC patients. In this article, we report the results of a phase I clinical trial approved by the CFDA (2009/04/03, CFDA 2009 L02105), intended to demonstrate the safety, immunogenicity and efficacy of Hu-rhEGF-rP64k/Mont in patients with advanced NSCLC.

## Methods

### Study design

This monocentric, open label dose-escalation phase I clinical trial enrolled 21 patients with NSCLC, in the National Cancer Center in Beijing, China. All participants were informed about the study and potential risks and required to provide written informed consent prior to undergoing study-related procedures.

A traditional 3 + 3 dose escalation design was implemented [12]. Successive cohorts of patients (3 patients/cohort) were each started on a fixed dose of the vaccine Hu-rhEGF-rP64k/Mont. Patients were assigned sequentially to one of four dose-escalation cohorts. The protocol specified 0.6 mg (group A) of the vaccine on days 4, 18, 32, 46 and 76 (5 vaccine dose), for the first cohort. Successive cohorts were given doses of 1.2 mg (group B), 1.8 mg (group C), 2.4 mg (group D) the same dose intervals. The dose groups were determined on the basis of the vaccine’s safety information available from previous clinical studies [8, 13, 14]. Each vaccine dose was administered at 1 injection site, deltoid region (group A), 2 injection sites, deltoids regions (group B), 3 injection sites, deltoids regions and gluteus region (group C) and 4 injection sites, deltoids and gluteus regions (group D).

A DLT was defined as any Grade 3 or 4 adverse event (AE) using the CTCAE 4.03 that was possibly drug-related. CTCAE 4.03 Grade 3 is a severe AE and Grade 4 is a life-threatening or disabling AE. Such events interfere with activities of daily living and include: skin toxicity, diarrhea or antidiarrheal therapy, vomiting at same grade for > 4 days despite aggressive antiemetic therapy, central nervous system, lung or renal toxicity or elevation of liver transaminases or bilirubin lasting more than 1 week. The number of patients who experienced dose-limiting toxicities (DLTs) was assessed at each dose level. If no DLTs were observed for 4 weeks after administration of the last dose of the vaccine, a new cohort of 3 patients was enrolled at the next planned dose level. If DLTs were observed in 1 patient in the cohort, another 3 patients were treated with the same dose level.

The maximum-tolerated dose (MTD) was defined as 1 dose level below the dose in which DLTs were observed in > 33% of the participants. That is, if DLTs were observed in at least 2 of 3 participants, the MTD was determined to be the dose administered to the previous cohort. Similarly, in a cohort of 6 participants, 3 of 6 participants would have to experience DLTs to determine the MTD.

Toxicities were graded using the Common Terminology Criteria for Adverse Events Version 4.03 (CTCAE 4.03) [15]. Health status assessments, including physical exams (vital signs, height, weight, ECOG score, skin of injection site, et al.), complete blood chemistry, electrocardiography was performed at baseline and post vaccination. Chest X-ray, computed tomography (CT) scan, and abdominal ultrasound were conducted at 46 days ±1 week and at 106 days ±1 week to assess clinical response according the Response Evaluation Criteria in Solid Tumors, version 1.1 (RECIST1.1). The objective response rate includes complete response (CR), partial response (PR), stable disease (SD), and progressive disease (PD). Disease control rate (DCR) was defined as CR + PR + SD.

Blood samples were collected at baseline (pre-treatment, day 1) and on days 18, 32, 46, 76 and 106 days post-dose for serum EGF concentration and anti-EGF antibody titers detection. Anti-EGF antibody titers were measured through a validated enzyme linked immunosorbent assay (ELISA), described in previous studies [8, 10] and EGF concentration in serum was measured with a commercial ELISA (Quantikine EGF; R&D Systems Inc., Minneapolis, MN). The detection was conducted by the Beijing Eastern Biotech, Co., Ltd.

The protocol and informed consent documents were reviewed and approved by the hospital human subjects review board and the study was performed in accordance with the Declaration of Helsinki.

### Patient eligibility

#### Inclusion criteria

Adult patients, regardless of gender, aged from 18 to 70 years with histologically or cytologically confirmed NSCLC at stages IIIB or IV and Eastern Cooperative Oncology Group (ECOG) performance status (PS) of 0 to 2 were eligible for the study. All patients received 4 to 6 cycles of platinum-based first-line chemotherapy, from 4 to 8 weeks, and were evaluated as SD or PR after the first line chemotherapy.

Patients were required to have at least 1 measurable lesion (lesions were defined by Response Evaluation Criteria in Solid Tumors (RECIST, 1.1) [16]. If patients have ever received radiotherapy, the disease in the field of radiotherapy cannot be the target lesions and additional requirements were adequate organ function and CBC results and life expectancy of at least 3 months”. Women participants should have negative pregnancy test results. All the participants should take contraception measures during the study period. Participants should sign informed consent before the study.

#### Exclusion criteria

Exclusion criteria included patients receiving immunosuppressant drugs 1 month before study, or receiving other immunotherapy 3 months before the study and patients receiving corticosteroid within 1 month before the research (including oral, intramuscular or intravenous injection). Inhaled corticosteroid for respiratory insufficiency or for local use were not excluded).

Patients with uncontrolled epilepsy seizure; central nervous system dysfunction or cognitive ability lose due to psychosis; CNS metastasis (confirmed or suspicious); chronic alcohol or drug abuse within 6 months before the research; pregnancy or lactation or those who refused to take contraception measures during the study were excluded. Patients with a history of severe allergy or allergic constitution; splenectomy; autoimmune disease or secondary immunodeficiency (such as AIDS, et al.); secondary malignancy within 5 years (except for basal cell carcinoma of the skin or intraepithelial carcinoma of the cervix) were also excluded.

### Treatment schedule

The therapeutic cancer vaccine is composed of hu-rhEGF conjugated to the carrier protein, r-P64K, and emulsified with the adjuvant Montanide ISA 51 VG, just prior to administration to patients. Hu-rhEGF-rP64k conjugate injection solution (batch number: 121005, 121006, 121203) and Montanide ISA 51 VG (batch number: 501101, 501201) were mixed in equal parts (0.8 mL of rhEGF/rP64k conjugate + 0.8 mL of Montanide ISA 51 VG). Hu-rhEGF-rP64k conjugate injection solution and the adjuvant were produced by the Center of Molecular Immunology (Havana, Cuba). CTX (Cyclophosphamide) injection solution (batch number: 04111001) was provided by BIOTECH PHARMA Co., Ltd.

Four to six weeks after finishing first-line chemotherapy, patients were assigned to the group of treatment with the vaccine. All patients received a low-dose of cyclophosphamide intravenously (200 mg/m2) on day 1. The vaccine was administered intramuscular at different dose levels: 0.6 mg (group A), 1.2 mg (group B), 1.8 mg (group C), and 2.4 mg (group D) on days 4, 18, 32, 46 and 76 (5 vaccine doses was administered for each patients. Monthly vaccination was administered according to the best benefits of patients and their wishes, and until the disease progressed or intolerable toxicity occurred.

### Study objectives

The primary aims of the study were safety evaluation, to establish the MTD of Hu-rhEGF-rP64k vaccine in NSCLC patients and the immunological response. The secondary outcome was clinical response assessment in NSCLC treated with Hu-rhEGF-rP64k vaccine. Objective Response Rate (CR + PR) and Disease Control Rate (CR + PR + SD) were the standards of assessment.

## Results

### Patients’ disposition

**Fig. 1:**
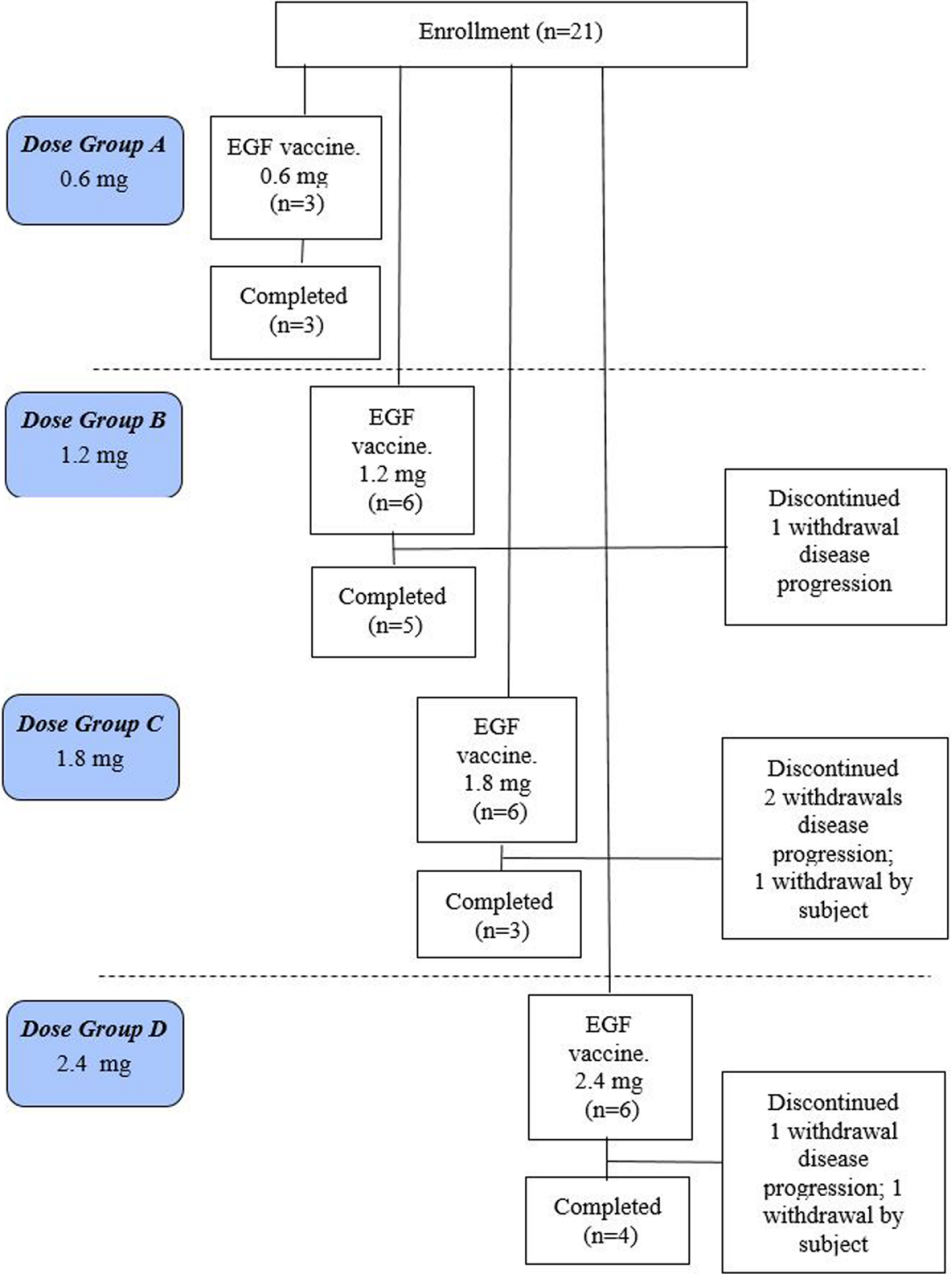
Flow Diagram: Enrollment, Assignment and Retention of Study Participants. 21 patients with non-small cell lung cancer were enrolled. Four fixed-dose groups (0.6 mg, 1.2 mg, 1.8 mg, and 2.4 mg) were set. Three patients were allocated to the 0.6 mg dose group, and received injection on days 4, 18, 32, 46 and 76 (5 vaccine dose). Six patients allocated to the 1.2 mg, 1.8 mg, 2.4 mg dose group separately and received the injection on the same dose intervals

A total of 21 patients were enrolled between April 10, 2012, and May 22, 2013, for four dose levels. The procedure of enrollment, assignment and retention of study participants are carried out according to the CONSORT statement [17] and are described in Fig. 1.

### Participants

Participant characteristics are listed in Table 1. Among the included patients, 12 were male while 9 were female, and median age was 54 (37~ 66). Median height was (167.29 ± 6.91) (158~ 180) cm. Median weight was (70.64 ± 12.56) (52~ 98) kg. Median Body Surface Area (BSA) was (1.78 ± 0.17) (1.54~ 2.10) m2. ECOG score: 5 patients scored 0 (23.81%) and 16 patients scored 1 (76.19%). According the histology, 2 cases were squamous cell carcinoma while 19 cases were adenocarcinoma.

**Table 1:** Baseline demographics and disease characteristic of the patients

Characteristic	Dose groups			
	A (0.6 mg)	B (1.2 mg)	C (1.8 mg)	D (2.4 mg)
Age (years), median	54.00	56.50	46.50	58.00
Gender *n* (%)				
Female	0(0.00%)	2(33.33%)	4(66.67%)	3(50.00%)
Male	3(100.00%)	4(66.67%)	2(33.33%)	3(50.00%)
Height (cm), Mean (SD)	171.33 (6.35)	167.17 (5.31)	163.67 (6.09)	169.00 (8.99)
Weight (kg), mean (SD)	70.67 (16.92)	72.17 (11.02)	67.67 (16.72)	72.08 (9.97)
BSA (cm2), Mean (SD)	1.82 (0.24)	1.80 (0.14)	1.70 (0.18)	1.82 (0.17)
ECOG^a^ PFS *n* (%)				
0	1(33.33%)	2(33.33%)	1(16.67%)	1(16.67%)
1	2(66.67%)	4(66.67%)	5(83.33%)	5(83.33%)
Histological type *n* (%)				
Squamous	1(33.33%)	1(16.67%)	0(0.00%)	0(0.00%)
Adenocarcinoma	2(66.67%)	5(83.33%)	6(100.00%)	6(100.00%)
First-line chemotherapy with cisplatin/carboplatin-based combination				
No. of Cycles (SD)	5.33 (0.94)	6.67 (1.15)	4.87 (1.21)	5 (1.53)
Reaction to Chemotherapy, PR/SD^b^	2/1	3/3	1/5	2/4

^a^*ECOG* Eastern Cooperative Oncology Group, *PFS* performance status

^b^*PR/SD* Partial Response/Stable Disease

The presence of mutations of the EGFR and KRAS were evaluated preliminary in 5 patients, K-RAS was not mutated and among them 2 patients have EGFR mutations (one has EGFR deletion 19 mutation and the other has EGFR deletion 21 mutation) and in one patient, EGFR mutation was no present. Since EGFR is constitutively activated in tumors with mutations at the intracellular domain, EGF binding will not be required for signal transduction, for this reason the evaluated vaccine would be effective in patients lacking EGFR mutations. In order to gather more information on the relevance of the individual tumor biology, additional translational studies should be included in the approaching trials, assessing the actual correlation between EGFR mutations and efficacy of the vaccine.

All patients (21) received at least one vaccine dose, 6 subjects (1 patient from group A 0.6 mg, 4 patients from group C 1.8 mg and 1 patient from group D 2.4 mg) received less than 5 doses and the rest 15 patients received 5 or more doses of vaccine. Three patients (2 patients from group A 0.6 mg and 1 patient from group C 1.8 mg) received more than 14 vaccine doses (1-year vaccination), 1 patient from group C 1.8 mg was vaccinated 26 times (2-years vaccination).

### Primary endpoints outcomes

#### Safety profile

In order to determine the primary endpoint, MTD, the number of participants who experienced DLTs was assessed at each dose level. According to the CTCAE 4.03, a DLT was any grade 3 non-hematological toxicity or grade 4 hematological toxicity that was possibly drug-related. CTCAE 4.03 Grade 3 is a severe AE and Grade 4 is a life-threatening or disabling AE. Such events interfere with activities of daily living. The MTD is defined as the dose level below the dose at which > 33% of participants experienced a DLT. The MTD analysis population consisted of all participants who received at least one dose of the vaccine.

No DLTs were observed by patients receiving the 4 doses levels. One patient discontinued the treatment due to disease progression among the three participants receiving 1.2 mg dose, thus three more participants were added to the cohort of which none experienced a DLT. Two patients’ withdrawals due to disease progression and 1 patient voluntary withdrawal among the participants receiving 1.8 mg vaccine dose, thus three more participants were added to the cohort of whom none experienced a DLT. One patient withdrawal due to disease progression and 1 patient voluntary withdrawal among the participants receiving 2.4 mg vaccine dose, thus three more participants were added to the cohort of whom none experienced a DLT.

The safety of vaccine was summarized by the number of patients experiencing any adverse event(s), serious and non-serious, which were collected by systematic assessment using terms from the CTCAE 4.03. Adverse events (AE) were reported in 14 cases (66.67%, 48 case-times) among all participants in all 4 cohorts. Ten patients have grade 1 adverse events and three patients have grade 2 adverse events, from them 1 patient in Group A, has WBC decrease; 1 patient in Group B has temporary amaurosis and 1 patient in Group C with erythema, itching and pain at the injection site. Only one patient has grade 3 AE, neutrophil count decreased, which was not related to the vaccine but to the previous chemotherapy. The summary of adverse events is presented in Table 2.

**Table 2:** Adverse events reported during the study

Adverse events	Group A - 0.6 mg		Group B - 1.2 mg		Group C - 1.8 mg		Group D - 2.4 mg	
	*n* = 3		*n* = 3		*n* = 3		*n* = 3	
	AEs	Patients	AEs	Patients	AEs	Patients	AEs	Patients
White blood cell decreased	1	1	1	1	1	1	2	1
ALT increased	1	1	0	0	1	1	0	0
Neutrophil count decreased	0	0	0	0	0	0	1	1
AST increased	0	0	0	0	1	1	0	0
Hemoglobin increased	1	1	0	0	0	0	0	0
Headache	0	0	0	0	3	3	0	0
Dizziness	4	2	4	3	5	3	0	0
Temporary amaurosis	0	0	1	1	0	0	0	0
Cough	0	0	0	0	1	1	0	0
Upper respiratory infection	1	1	0	0	0	0	0	0
Hyperhidrosis	0	0	0	0	1	1	0	0
Fever	0	0	1	1	1	1	0	0
Tremors	0	0	2	1	2	1	0	0
Face flushing	3	1	0	0	0	0	0	0
Injection site erythema	0	0	0	0	2	1	0	0
Injection site itching	0	0	0	0	1	1	0	0
injection site pain	3	1	2	2	0	0	0	0
Nausea	0	0	0	0	1	1	0	0
Total	14		11		20		3	

The most frequents adverse event were dizziness (27.1%); injection-site pain (10.4%); tremors (8.3%); headache (6.25%); face flushing (6.25) and injection-site erythema (4.2%). The majority of the adverse events (90%) were grade 1 or 2. Only three cases of grade 3 adverse events, in the 2.4 mg dose level (Group D), were reported, none of them were related to the vaccine. These severe adverse events (SAEs) were: 1 case of grade 3 leukopenia, 1 case of grade 4 neutropenia and 1 case of anal fistula (grade 3), where hospitalization was indicated and the patient was submitted to surgery and recovered from the AE. This last adverse event was reported as non-related SAE. None patients were withdrawn the study due to adverse events.

By the date of submission, 1 case of anemia had been improved, and in 1 case of leukopenia the condition is still ongoing. All other adverse events have been recovered. None patient was dropout of the research due to adverse events. No death cases during the research period.

#### Immunological response: Serum EGF concentration and anti-EGF antibodies detection

Serum EGF and anti-EGF antibody titers were evaluated in 21 patients before and after vaccination. Blood samples were collected at baseline (pre-treatment, day 1) and on days 18, 32, 46, 76 and 106 days post-vaccination. Anti-EGF antibody titers were measured through an ELISA. EGF concentration in serum was measured with a commercial ELISA (Quantikine; R&D Systems Inc., Minneapolis, MN).

**Fig. 2:**
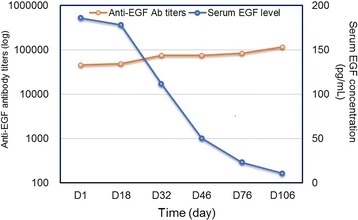
Kinetics of the anti-EGF antibody titers and serum EGF concentration in patients. A significant inverse correlation was observed (Spearman rho = − 1.000, *p* < 0, 01) between the anti-EGF antibody titers and serum EGF concentration in patients treated with the vaccine

The kinetics of the geometric mean of anti-EGF antibody titers and serum EGF concentration in patients are described in Fig. 2. As showed in the figure, a significant inverse correlation was observed (Spearman rho = − 1.000, *p* < 0, 01) between the anti-EGF antibody titers and serum EGF concentration in patients treated with the vaccine.

### Secondary endpoints

#### Clinical response evaluation

The first clinical response evaluation was done at 46 days ±1 week and the second evaluation was done at 106 days ±1 week. After the first response assessment (*n* = 20), none patients achieved CR or PR, and 15 patients (75%) were on SD. After the second response evaluation (*n* = 15), no patients had achieved objective response (CR or PR), while 12 (80%) patients had SD and the remaining three (20%) patients had progressive disease. DCR for each dose group were: 100% for 0.6 mg dose group; 60% for 1.2 mg dose group; 66.67% for 1.8 mg dose group and 100% for 2.4 mg dose group. Detailed information is presented on Table 3.

**Table 3:** Clinical response to Hu-rhEGF-rP64k vaccine

First evaluation [*n* (%)] Day 46					
Response	Group A–0.6 mg	Group B- 1.2 mg	Group C-1.8 mg	Group D-2.4 mg	Total
	*n* = 3	*n* = 6	*n* = 5	*n* = 6	*n* = 20
CR	0(0%)	0(0%)	0(0%)	0(0%)	0(0%)
PR	0(0%)	0(0%)	0(0%)	0(0%)	0(0%)
SD	3(100%)	4(67%)	3(60%)	5(83%)	15(75%)
PD	0(0%)	2(33%)	2(40%)	1(17%)	5(25%)
Second evaluation [n (%)] Day 106					
Response	Group A–0.6 mg	Group B- 1.2 mg	Group C-1.8 mg	Group D-2.4 mg	Total
	*n* = 3	*n* = 5	*n* = 3	*n* = 4	*n* = 15
CR	0(0%)	0(0%)	0(0%)	0(0%)	0(0%)
PR	0(0%)	0(0%)	0(0%)	0(0%)	0(0%)
SD	3(100%)	3(60%)	2(67%)	4(100%)	12(80%)
PD	0(0%)	2(40%)	1(33%)	0(0%)	3(20%)

## Discussion

Worldwide lung cancer is the main cause of cancer-related death [1]. Among the cases of lung cancer, NSCLC histology prevails, representing between 75 and 80% of lung tumors [2, 3]. Improvements in the treatment of advanced disease with first-line and subsequent therapies are allowing longer survival, disease control and superior quality of life for the patients. Four to six cycles of platinum-based chemotherapy has been the standard first line for the treatment of unselected patients with this cancer subtype. Second-line chemotherapy may include pemetrexed in non-squamous cancer and docetaxel or erlotinib (or both) in all types of NSCLC. NSCLC management has positively evolved from the emergence of immunotherapy, currently positioned as second line therapy [18]. Antitumor activity, long lasting response and survival increases in advanced NSCLC patients has been demonstrated with the use of anti-PD-1 passive immunotherapy [19], leading to regulatory approval of an anti-PD-1 drug, nivolumab (Opdivo; Bristol-Myers Squibb) [20] for the treatment of metastatic squamous non–small-cell lung cancer (NSCLC) in patients who have progressed on a platinum-based chemotherapy.

Active cancer immunotherapy is also gaining importance in management of advanced stage. Hu-rhEGF-rP64k vaccine is a therapeutic modality that exerts its anti-cancer activity by targeting the immune system, specifically the self-molecule Epidermal Growth Factor (EGF). The activation of EGF receptor, blocks apoptosis, stimulates cell proliferation, induces malignant transformation, angiogenesis and metastasis [21]. In Hu-rhEGF-rP64k vaccine, the human EGF protein is chemically conjugated to P64K protein, which is a highly immunogenic molecule from Neisseria meningitides, additionally the conjugate is emulsified in the Montanide ISA51 VG adjuvant, enhancing the immunogenicity and promoting the generation of higher titers of anti-EGF antibody titers [22]. The antibodies generated by Hu-rhEGF-rP64k vaccination produced a deprivation of circulating EGF and avoid the activation of the receptor, preventing the initiation of the intracellular signaling cascade. Clinically, the vaccine produces disease stabilization and a prolongation of survival [6, 23].

The preclinical studies found that Hu-rhEGF-rP64k vaccine is immunogenic, well tolerated and has anti-tumor activity [24–27]. The Phase I to Phase III clinical trials have shown overall survival benefits in addition to an improvement of the quality of life in NSCLC patients [6–9, 11, 22]. It has been demonstrated in NSCLC patients treated with Hu-rhEGF-rP64k vaccine in multiple clinical trials, that high anti-EGF antibodies titers followed by the consequent reduction of serum EGF levels correlates with a significant overall survival benefit for subgroups of patients, especially for those patients with high baseline levels of serum EGF [11]. No serious safety concerns have arisen with the use of Hu-rhEGF-rP64k vaccine and to date the vaccine has shown a mild and very well tolerated adverse event profile.

The primary endpoint in this study was safety. Repeated administration Hu-rhEGF-rP64k vaccine did not result in any dose-limiting toxicities or serious adverse events related to vaccine treatment. The vaccine doses in the four groups (0.6 mg; 1.2 mg; 1.8 mg and 2.4 mg) were well tolerated. MTD was not established in this study. Similar to previous clinical trials [13], the most frequent adverse events were grade 1–2 dizziness, injection-site pain and erythema, tremors and headache. There were no deaths attributed to the vaccine treatment in this clinical trial. The data gathered from this study confirms that under the condition of the study, rhEGF-rP64k vaccine is safe in patients with advanced NSCLC and prolonged vaccination schedule could be administered without inducing severe adverse effects.

Immunological response was also a primary endpoint of this clinical trial. Each dose of Hu-rhEGF-rP64k vaccine was administered from 1 to 4 injections sites, by intramuscular route. The selection of the clinical immunization schedule was based on the previous non-clinical and clinical studies, consisting of an induction phase (to induce maximum antibodies titers) of four bi-weekly immunizations followed by a maintenance phase consisting of monthly re-immunization to maintain the anti-EGF antibodies titers developed during the induction stage [11]. In this phase I trial this dose schedule was effective to induced the production of anti-EGF antibodies that correlated inversely with the EGF concentration. The association between the immunological response and the efficacy parameters was not evaluated in this trial but should be addressed in the subsequent trials since the data of previous studies suggest that a significant survival benefit is achieved mainly in patients having good antibody response after induction period and high EGF concentration after front line chemotherapy [11].

After Hu-rhEGF-rP64k vaccination, 80% of the patients had SD, which confirm that the vaccine after first-line chemotherapy can get high disease control rate. The anti-cancer activity of this novel vaccine target the immune system, inducing an increase in the patient’s anti-Epidermal Growth Factor (EGF) antibody titers and reduction of the circulating EGF [13]. The effect of the vaccine on the tumor is not a direct one, it is manifested through the impact on the patient’s immune response and may not cause important tumor regression but may significantly slow tumor growth and a final positive impact on survival is achieved.

The data collected from this phase I clinical trial demonstrated that Hu-rhEGF-rP64k vaccination in advanced NSCLC patient is safe and confirm the expected effect on the immune system and on the tumor of the included patients. A new clinical trial to confirm the impact of this vaccine on the survival of patients should be performed.

## Conclusion

Hu-rhEGF-rP64k/Mont elicited a valuable immune response, with good safety profile assuring further clinical development of the vaccine in this population to further confirm the potential benefits on survival.

## Abbreviations

AE: Adverse event
BSA: Body surface area
CR: Complete response
DCR: Disease control rate
DLT: Dose-limiting toxicity
ECOG: Eastern Cooperative Oncology Group
EGF: Epidermal growth factor
EGF-R: Epidermal growth factor receptor
ELISA: Enzyme linked immunosorbent assay
MTD: Maximum-tolerated dose
NSCLC: Non-small cell lung cancer
PD: Progressive disease
PR: Partial response
PS: Performance status
rhEGF: Recombinant human epidermal growth factor
rP64K: Recombinant P64K
SAE: Severe adverse event
SD: Stable disease
